# Dioscin inhibits stem-cell-like properties and tumor growth of osteosarcoma through Akt/GSK3/β-catenin signaling pathway

**DOI:** 10.1038/s41419-018-0363-x

**Published:** 2018-03-01

**Authors:** Weihai Liu, Zhiqiang Zhao, Yongqian Wang, Wuguo Li, Qiao Su, Qiang Jia, Jiajun Zhang, Xuelin Zhang, Jingnan Shen, Junqiang Yin

**Affiliations:** 1 Department of Musculoskeletal Oncology, The First Affiliated Hospital of Sun Yat-sen University, Guangzhou, China; 2 Department of Animal Experiment Center, The First Affiliated Hospital of Sun Yat-sen University, Guangzhou, China; 3 Guangzhou City Polytechnic, Guangzhou, China

## Abstract

Osteosarcoma is the most common primary bone tumor in children and adolescents. Many patients with osteosarcoma always develop drug resistance to current chemotherapy regimens, which induces a poor prognosis. And cancer stem cells (CSCs) have been reported to possess the properties to self-renew and maintain the phenotype of tumor, which may lead to clinical treatment failure. Thus, it is an urgent task to develop several potentially useful therapeutic agents, which could target CSCs in osteosarcoma. This study aims to clarify the in vitro and in vivo anti-osteosarcoma effects of dioscin, the primary component derived from *Discorea nipponica Makino*, and its molecular mechanism of action. In this study, all the ten human osteosarcoma cell lines were sensitive to dioscin treatment in a dose- and time-dependent manner. Dioscin inhibits proliferation and induces cell cycle arrest as well as apoptotic cell death in osteosarcoma cells. More importantly, oral administration of dioscin (60 mg/kg) showed significant therapeutic effect on osteosarcoma growth without obvious side effects in vivo. In addition, dioscin possesses the ability to suppress stem-cell-like phenotype of osteosarcoma cells. Mechanistically, dioscin inhibits osteosarcoma stem-cell-like properties and tumor growth through repression of Akt/GSK3/β-catenin pathway. Moreover, β-catenin expression in osteosarcoma patients was associated with clinical prognosis. Conclusively, the present study provides comprehensive evidence for the inhibition of dioscin on osteosarcoma stem-cell-like properties and tumor growth through repression of Akt/GSK3/β-catenin pathway, which suggests dioscin as a promising therapeutic regimen. And β-catenin may be a potential therapeutic target as well as a significant prognostic marker for osteosarcoma patients in clinic.

## Introduction

Osteosarcoma is the most common primary bone tumor, which mainly affects children and adolescents^1^. The prognosis of patients with osteosarcoma can be improved with the combination of surgery and chemotherapy, and the 5-year survival rate has reached 60−70% in the recent years^2^. However, a considerable number of patients either are not sensitive to chemotherapy or develop drug resistance with current chemotherapy regimens^3^. Therefore, one strategy to avoid chemo-resistance and improve clinical outcomes is to identify effective therapeutic agents that can increase drug-response rates.

It has been a long history for agents derived from natural sources to be used for clinical treatment, which shows multiple biological activities, such as anti-inflammation and anti-tumor properties^4–7^. One of the most successful examples for natural agents is artemisinin (qinghaosu), which is proved to possess significant antimalarial effect by Youyou Tu who was awarded the 2015 Nobel Prize in Physiology or Medicine for her discoveries concerning a novel therapy against malaria.

*Discorea nipponica Makino* is a traditional Chinese medicinal herb and has been used as an antitussive and antiasthmatic agent for a long period of time. Dioscin, a kind of steroidal saponin extracted from *Discorea nipponica Makino*, has been reported to have many biologic effects, including attenuating renal ischemia/reperfusion injury, anti-inflammatory, and anti-allergic properties^8,9^. Recently, dioscin has shown significant anti-tumor activity in several types of cancer^10,11^. However, very little is known about the effects of dioscin on osteosarcoma, and the molecular mechanisms of its anti-tumor activity remain unclear. Therefore, the aim of our study was to investigate the in vitro and in vivo anti-osteosarcoma effects of dioscin and to clarify its molecular mechanism of action, which can help confirm its potential as a therapeutic agent in the treatment of osteosarcoma patients. Moreover, identifying the targets of dioscin may also help provide valuable biomarkers as prognostic predictive factors for osteosarcoma patients.

## Materials and methods

### Chemicals and reagents

Dioscin (MW: 869.04 Da; >98% purity) was extracted from *Discorea nipponica Makino*. And 3-(4,5-dimethylthiazole-2-yl)-2,5-diphenyl tetrazolium bromide (MTT), adriamycin were purchased from Sigma-Aldrich. ICG-001 and XAV-939 were purchased from Selleck Chemicals.

### Cell culture

The human osteosarcoma cell lines, U2OS, HOS, MNNGHOS, 143B, MG63, SJSA1, and G292, were obtained from the American Type Culture Collection (ATCC). The ZOS and ZOS-M cell lines have been described previously^12^. U2OS/MTX300 cells, a methotrexate-resistant derivative of the U2OS human osteosarcoma cell line, were provided by Dr. M. Serra (Istituti Ortopedici Rizzoli, Bologna, Italy), which was continuously cultured in the presence of 300 μg/l MTX^13^. All the other cells were cultured in DMEM (Gibco, Grand Island, NY, USA) and supplemented with 10% fetal bovine serum (Gibco, Grand Island, NY, USA), penicillin (10,000 U/l), and streptomycin (100 mg/l) at 37 °C in a 5% CO_2_ humidified incubator.

### MTT assay

A total of 2000 cells were plated in 96-well flat-bottom plates and exposed to dioscin at different concentrations in a final volume of 180 μl. At the indicated times, 20 μl of 5 mg MTT/ml in PBS were added to each well, and the plates were incubated at 37 °C for 4 h. After removal of the medium, 150 μl DMSO was added to each well to dissolve the formazan crystals. Absorbance at 490 nm was determined using a microplate reader.

### Cell cycle analysis

Cells were treated with 2.5 μm dioscin at 37 °C in a 5% CO_2_ incubator for 24 h and were subsequently collected and analyzed using a Cytomics FC 500 instrument (Beckman Coulter) after propidium iodide staining. The percentage of cells in the different phases of the cell cycle were determined with cell-cycle analysis software.

### Annexin V/PI staining assay for apoptosis

The cells were treated with 2.5 μm dioscin for 48 h and were subsequently collected, washed twice with PBS, and incubated with Annexin V-FITC and propidium iodide for 30 min in the dark. Cell apoptosis was analyzed using FACScan (Becton Dickinson, Franklin Lakes, NJ, USA), and CellQuest software was used to analyze the original data.

### Colony-formation assay

Cells were seeded in triplicate at a density of 800 cells/well in six-well flat-bottom plates with 2 ml DMEM containing 10% fetal bovine serum. Cells were incubated with or without dioscin treatment for 48 h and then cultured for 10 days at 37 °C in a 5% CO_2_ incubator. Cell colonies were fixed in methanol and stained with crystal violet. The number of colonies that contained >50 cells was counted under microscope.

### Hoechst 33258 staining

Cells were seeded at 50% density in six-well flat-bottom plates, and after overnight incubation the cells were treated with vehicle or dioscin for 24 h. Then, the cells were fixed and then stained with the Hoechst 33258 dye. The morphologic changes and characters of apoptosis were observed using laser scanning confocal microscope (Olympus FV1000).

### Sarcosphere-formation assay

Sarcosphere-formation assay was performed as previously described^14^. And the secondary sarcosphere-formation assay was performed without further treatment after compounds treatment in primary sarcosphere-formation assay.

### Immunohistochemistry analysis

For immunohistochemistry, sections were incubated with antibody against Bcl-2 (1:100, #ab32124, Abcam), Ki-67 (1:100, #ab16667, Abcam), proliferating cell nuclear antigen (PCNA) (1:100, #ab92552, Abcam), β-catenin (1:200, #ab22656, Abcam), Gli1 (1:100, #ab151796, Abcam), NICD1 (1:100, #07-1232, Millipore, Billerica, MA, USA), phospho-Akt (Ser473) (1:100, #ab81283, Abcam), phospho-GSK3β (Ser9) (1:100, #ab75814, Abcam) at 4 °C overnight. Primary antibodies were detected by the Dako EnVision Kit (Dako, Glostrup, Denmark) according to the manufacturer’s protocol. The staining intensity was evaluated and scored by two independent pathologists. The extent of staining was scored as 0, 0% of cells stained; 1, 1–25% of cells stained; 2, 26–50% of cells stained; 3, 51–75% of cells stained; or 4, >75% of cells stained. Staining intensity was scored as 0, negative; 1, weak; 2, intermediate or 3, strong. The final staining score was defined as the product of the extent and intensity scores.

### Immunofluorescence analysis

After they were treated with vehicle or dioscin, cell samples were fixed using 4% paraformaldehyde solution for 15 min at room temperature and then extracted with buffer containing 0.5% Triton X-100 for 5 min. The cells were then incubated with primary antibodies against SOX2 (1:100, #ab92494, Abcam), β-catenin (1:100, # ab22656, Abcam), phospho-Akt (Ser473) (1:100, #ab81283, Abcam), phospho-GSK3β (Ser9) (1:100, #ab75814, Abcam) at 4 °C overnight. Next, samples were incubated with secondary antibody at room temperature for 1 h. Finally, nuclei were counterstained with DAPI. Immunofluorescence was detected using laser scanning confocal microscope (Olympus FV1000).

### RNA extraction and quantitative real-time PCR

After cells were treated with vehicle or dioscin, total cellular RNA was extracted using the TRIzol reagent (Invitrogen) according to the manufacturer’s instructions. The cDNA was synthesized by PrimeScript^TM^ RT reagent Kit (Takara), according to the manufacturer’s protocol. Real-time PCR amplification was performed using SYBR^®^ *Premix* (Takara) on Real-Time PCR System (ABI ViiATM7Dx). Primers are the following:

**Table:** 

Gene	Forward primer	Reverse primer
*P21*	TGTCCGTCAGAACCCATGC	AAAGTCGAAGTTCCATCGCTC
*SOX2*	TACAGCATGTCCTACTCGCAG	GAGGAAGAGGTAACCACAGGG
*OCT4*	GGGAGATTGATAACTGGTGTGTT	GTGTATATCCCAGGGTGATCCTC
*CD133*	AGTCGGAAACTGGCAGATAGC	GGTAGTGTTGTACTGGGCCAAT
*CD117*	CGTTCTGCTCCTACTGCTTCG	CCCACGCGGACTATTAAGTCT
*NANOG*	CCCCAGCCTTTACTCTTCCTA	CCAGGTTGAATTGTTCCAGGTC
*PPARD*	ACTGAGTTCGCCAAGAGCATC	ACGCCATACTTGAGAAGGGTAA
*AXIN2*	CAACACCAGGCGGAACGAA	GCCCAATAAGGAGTGTAAGGACT
*MMP7*	GAGTGAGCTACAGTGGGAACA	CTATGACGCGGGAGTTTAACAT
*HES1*	CCTGTCATCCCCGTCTACAC	CACATGGAGTCCGCCGTAA
*CCND3*	TACCCGCCATCCATGATCG	AGGCAGTCCACTTCAGTGC
*GLI1*	AACGCTATACAGATCCTAGCTCG	GTGCCGTTTGGTCACATGG
*HHIP*	TCTCAAAGCCTGTTCCACTCA	GCCTCGGCAAGTGTAAAAGAA
*GAPDH*	ACAACTTTGGTATCGTGGAAGG	GCCATCACGCCACAGTTTC

### Western blot analysis

The analysis was performed according to standard procedures described previously^15^. Antibodies against p21 (1:1000, #2947, CST), Cleaved PARP (1:1000, # ab32064, Abcam), Bcl-2 (1:1000, #ab32124, Abcam), Bcl-xL (1:500, #D120306, Sangon Biotech, Shanghai, China), Gli1 (1:1000, #ab151796, Abcam), NICD1 (1:500, #07-1232, Millipore, Billerica, MA, USA), β-catenin (1:1000, #8480, CST), Lamin B1 (1:2000, #66095-1-Ig, Proteintech), phospho-Akt (Ser473) (1:1000, #4060, CST), Akt (1:1000, #4691, CST), phospho-GSK3α/β (Ser21/9) (1:1000, #8566, CST), GSK3α/β (1:1000, #5676, CST), GAPDH (1:1000, #5174, CST), β-actin (1:1000, #5125, CST) were used.

### Animal studies

All studies were approved by the medical ethical committee of the First Affiliated Hospital of Sun Yat-sen University. And we completed the animal experiments according to the guidelines in Center of Experiment Animal of the First Affiliated Hospital of Sun Yat-sen University. Female nude mice, 4 weeks old, were purchased from Nanjing Biomedical Research Institute of Nanjing University. 143B cells (1×10^6^ cells in 200 µl PBS) were injected subcutaneously near the scapula of the nude mice. Eight days after injection, the mice were randomly separated into appropriate groups (vehicle, dioscin, ADM). Mice were treated with the vehicle or dioscin (60 mg/kg)^10^ by oral administration every day. ADM was injected intraperitoneally at a dose of 6 mg/kg once a week. Body weights of the mice as well as the length and width of the tumors were measured every 4 days, and the tumor volume was calculated using the formula *V* = 1/2 (width^2^ × length). All mice were killed at the end of the experiment, and the tumor weights were measured after careful resection. H&E staining of the mice organs was performed according to standard procedures.

### Receiver operating characteristic (ROC) curve analysis

Receiver operating characteristic (ROC) curve analysis was employed to define an optimal cutoff value for high β-catenin expression by using the 0, 1-criterion. The IHC score with the shortest distance from the curve to the point (0.0, 1.0), which represents both maximum sensitivity and specificity, was selected as the cutoff value^16^. Cases with the IHC scores high or equal to the cutoff value were defined as high β-catenin expression, while low β-catenin expression cases were those with scores below the cutoff value.

### Statistical analyses

All data were obtained from three independent experiments and expressed as the mean ± SD. The differences were analyzed using two-tailed Student’s *t*test or log-rank test (SPSS software 20.0), and a *p*-value of <0.05 was considered significant.

## Results

### Dioscin inhibits proliferation, induces cell cycle arrest, and apoptotic cell death in osteosarcoma cells

**Fig. 1:**
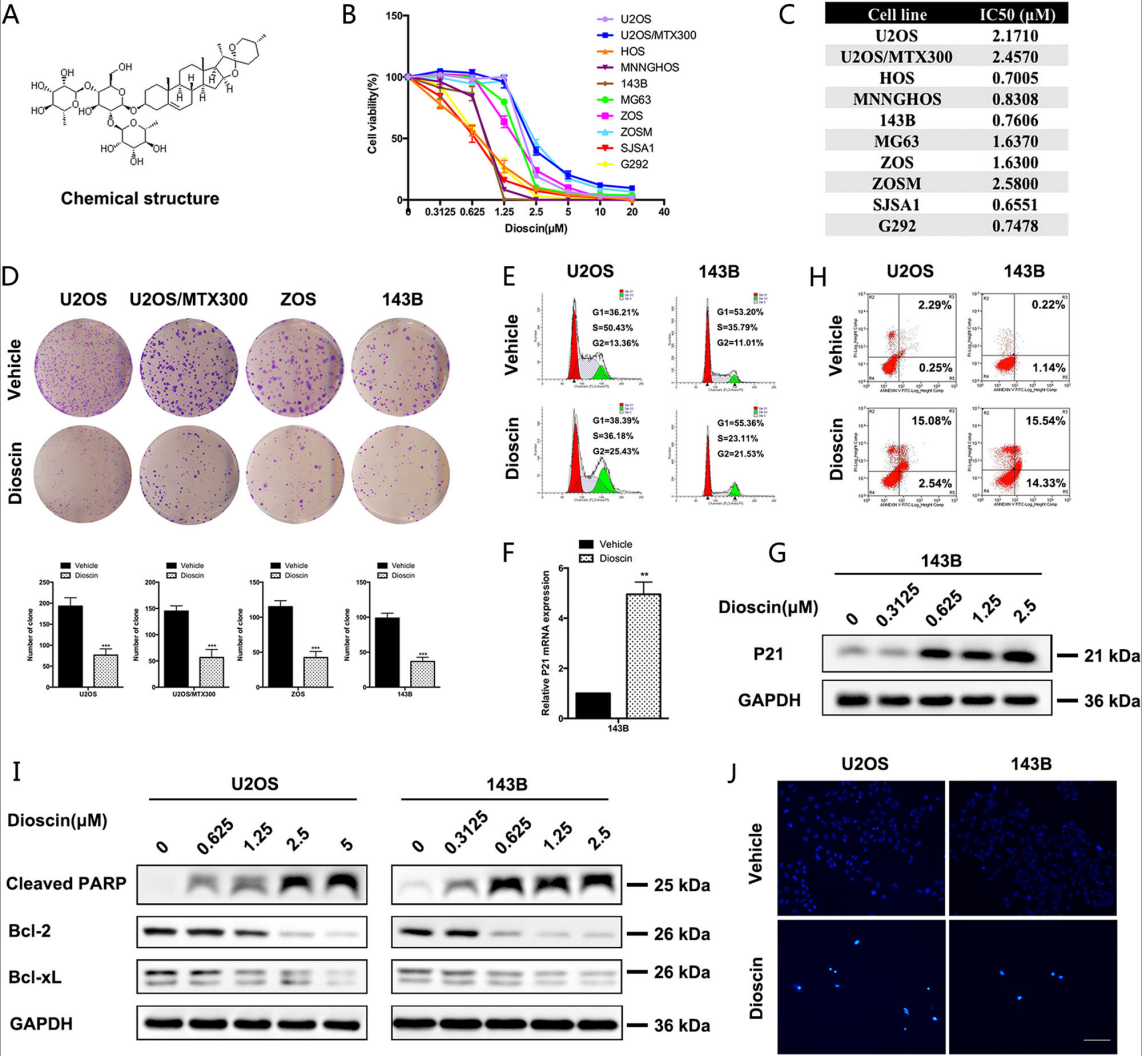
Dioscin inhibits proliferation and induces apoptotic cell death in osteosarcoma cells. **a** Chemical structure of dioscin. **b**, **c** Dioscin inhibits osteosarcoma cell (U2OS, U2OS/MTX300, HOS, MNNGHOS, 143B, MG63, ZOS, ZOSM, SJSA1, G292) viability in a dose-dependent manner. Osteosarcoma cells were treated with various concentrations of dioscin for 72 h, and the viability of cells was measured by the MTT assay. And the IC50 were calculated and shown. **d** Dioscin reduces colony formation of osteosarcoma cells. Colony-formation ability of osteosarcoma cells (U2OS, U2OS/MTX300, ZOS, 143B) was examined after dioscin treatment for 10 days. **e** Dioscin induces a G2/M phase arrest in osteosarcoma cells (U2OS, 143B). Cell-cycle distribution of osteosarcoma cells treated with vehicle or 2.5 μm dioscin for 24 h was detected and analyzed by flow cytometry. **f**, **g** Dioscin upregulates the expression of P21 at both mRNA and protein levels detected by qRT-PCR and western blot, respectively. **h** Annexin V/PI staining of osteosarcoma cells (U2OS, 143B) treated with vehicle or 2.5 μm dioscin for 48 h was detected and analyzed by flow cytometry. **i** Several apoptosis indexes (cleaved PARP, Bcl-2 and Bcl-xL) were detected by western blot. Dioscin induces cleavage of PARP as well as downregulation of Bcl-2 and Bcl-xL. **j** Hoechst staining showed brighter blue staining and typical morphological changes of apoptosis including the reduction of nuclear size and chromatin condensation in nuclear chromatin of U2OS and 143B cells after 2.5 μm dioscin treatment for 24 h. Scale bar, 100 μm. Data represent the mean ± SD of three independent experiments. **p < 0.01, ***p < 0.001 by two-tailed Student’s *t* test, SPSS 20.0

Dioscin is the major component derived from *Discorea nipponica Makino*, and its chemical structure (molecular weight, 869.04 Da) is shown in Fig. 1a. To investigate whether the proliferation of OS cells is sensitive to dioscin treatment, ten human osteosarcoma cell lines were treated with increasing concentrations of dioscin for 72 h. Dioscin reduced cell viability of all osteosarcoma cell lines in a dose- and time-dependent manner (Fig. 1b and Supplementary Figure S1A and B), with the calculated IC50 ranging from 0.6551 to 2.5800 μm (Fig. 1c). To further explore the suppression effect of dioscin on OS cell growth, we performed colony-formation assay and observed that the number and size of colony were greatly reduced in OS cells treated with dioscin compared with controls (Fig. 1d). Conclusively, these results indicate that dioscin suppresses the proliferation of OS cells in vitro.

To examine whether dioscin affects cell-cycle distribution, human osteosarcoma cell lines U2OS and 143B were treated with dioscin (2.5 μm for 24 h) followed by flow cytometry. And we observed a G2/M phase arrest (Fig. 1e). Moreover, we detected the expression of P21, a potent inhibitor of cell-cycle progression, and found that P21 was upregulated at both mRNA and protein levels after dioscin treatment (Fig. 1f, g). These results suggest that dioscin not only inhibited cell proliferation but also induced cell cycle arrest in OS cells.

### Dioscin induces apoptotic cell death in osteosarcoma cells

Besides proliferation suppression, we further found that dioscin efficiently induced apoptosis in OS cells. Annexin V/PI staining of OS cells showed that the portion of apoptotic cells increased significantly after 2.5 μm dioscin treatment (Fig. 1h). Furthermore, the pro-apoptotic effect of dioscin was indicated by the induced cleavage of PARP as well as downregulation of Bcl-2 and Bcl-xL (Fig. 1i). And the effect of dioscin on cell apoptosis was analyzed by Hoechst 33258 staining. Brighter blue staining and typical morphological changes of apoptosis such as the reduction of nuclear size and chromatin condensation were more easily observed in nuclear chromatin of U2OS and 143B cells after 2.5 μm dioscin treatment for 24 h (Fig. 1j). Collectively, these results indicate that dioscin promotes the apoptosis of osteosarcoma cells in vitro.

### Dioscin inhibits the growth of osteosarcoma xenografts in vivo

**Fig. 2:**
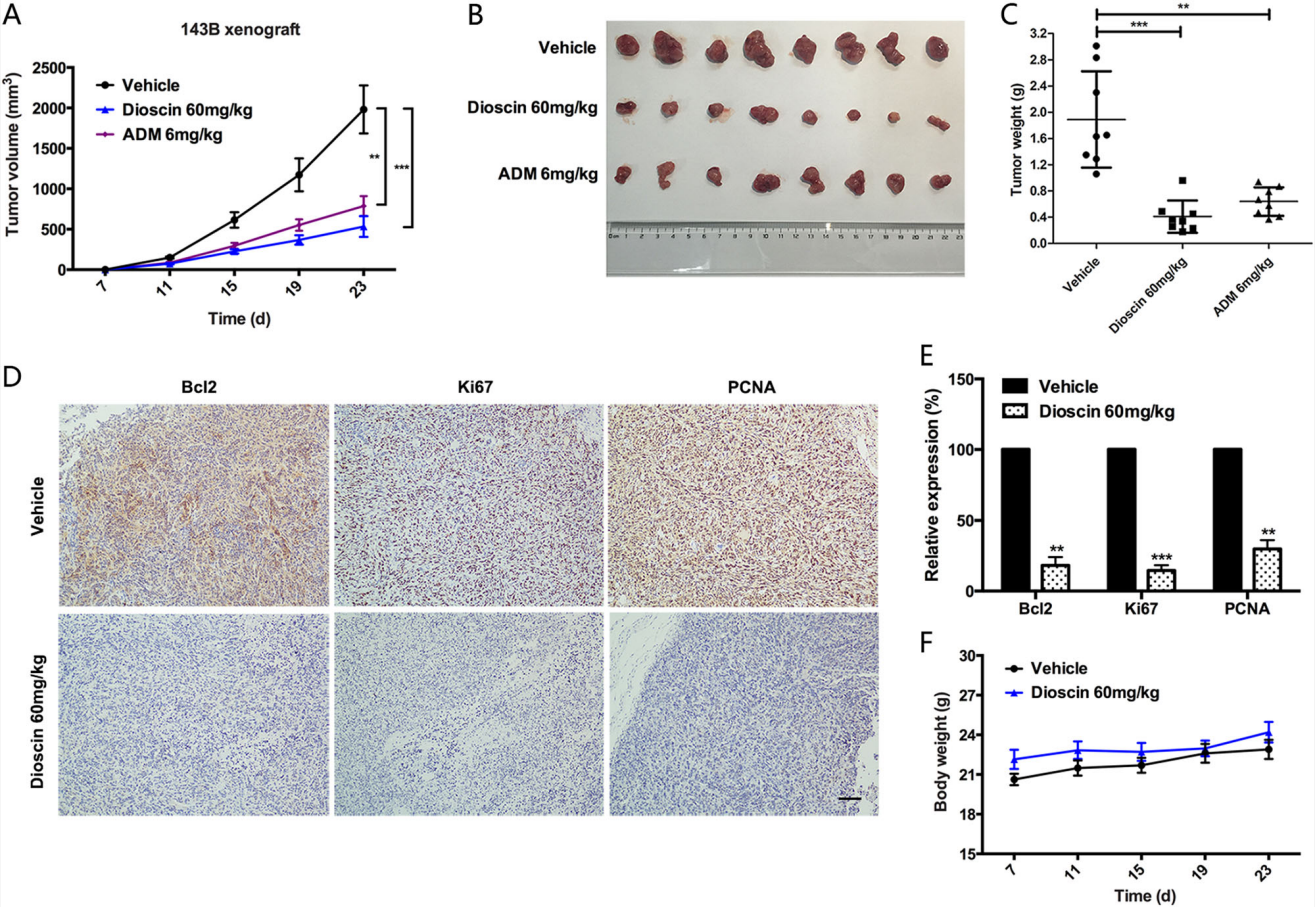
Dioscin inhibits the growth of osteosarcoma xenografts in vivo. **a**−**c** Examination of tumor volumes and weight to evaluate the effect of different treatments (Vehicle, Dioscin 60 mg/kg and ADM 6 mg/kg) on 143B cells in a xenograft model. Dioscin was given by oral administration at a dose of 60 mg/kg every day. ADM was injected intraperitoneally at a dose of 6 mg/kg once a week. Data represent the mean ± SD of tumor volume and weight of eight mice. **d** IHC staining of Bcl2, Ki67, and PCNA in mice tumor samples to examine the level of in vivo apoptosis induced by dioscin treatment and its inhibitory effect on osteosarcoma cell proliferation. Scale bar, 100 μm. **e** The intensity of IHC staining (Bcl2, Ki67, and PCNA) was scored. Dioscin 60 mg/kg significantly decreased the expression of Bcl2, Ki67, and PCNA in vivo. Data represent the mean ± SD of IHC staining scores of eight mice. **f** Effect of dioscin on the body weight of mice over the treatment time. Data represent the mean ± SD of body weight of eight mice. **p < 0.01, ***p < 0.001 by two-tailed Student’s *t*test, SPSS 20.0

The above experimental evidence has shown the inhibitory effect of dioscin on osteosarcoma in vitro. We sought to further explore whether dioscin could inhibit the growth of osteosarcoma in vivo. Firstly, 143B cells were subcutaneously injected into nude mice until a tumor volume of approximately 200 mm^3^. And then the mice were randomly separated into three groups (Vehicle, Dioscin 60 mg/kg and ADM 6 mg/kg). The Dioscin group received 60 mg/kg of dioscin every day by oral administration. The ADM 6 mg/kg group was set as a positive control. A significant tumor size reduction in mice treated with dioscin and ADM was observed at the termination of the study, which was reflected by the tumor growth curve. The mean volumes of the tumors were 1981.10 mm^3^ for the Vehicle group, 533.55 mm^3^ for the Dioscin group (*p* < 0.001), and 787.33 mm^3^ for the ADM group (*p* < 0.01) (Fig. 2a, b). The average tumor weights were 1.89 g for the Vehicle group, 0.41 g for the Dioscin group (*p* < 0.001), and 0.64 g for the ADM group (*p* < 0.01) (Fig. 2c). Although the *p* value was no less than 0.05, a greater decline in tumor volumes (*p* = 0.175) and tumor weights (*p* = 0.069) was observed in the Dioscin group than the ADM group, which suggested that dioscin may have a greater inhibitory effect on osteosarcoma than ADM, one of the currently used chemotherapy drugs.

Moreover, IHC assay for mice tumor samples was performed to examine the level of in vivo apoptosis induced by dioscin treatment and its inhibitory effect on osteosarcoma cell proliferation. And the results showed that the tumor treated with dioscin had a significant decrease in the expression of Bcl2, Ki67, and PCNA (Fig. 2d, e). In addition, there was no significant difference in the average body weight of the mice between the Vehicle group and the Dioscin group (Fig. 2f), and no obvious pathological changes were observed in the important organs (heart, liver, spleen, lung, and kidney), as detected by H&E staining (Supplementary Figure S1C).

Conclusively, these results demonstrate that dioscin possesses anti-tumor properties in human osteosarcoma cells, without severe toxicity and side effects in vivo.

### Dioscin suppresses stem-cell-like properties of osteosarcoma cells

**Fig. 3:**
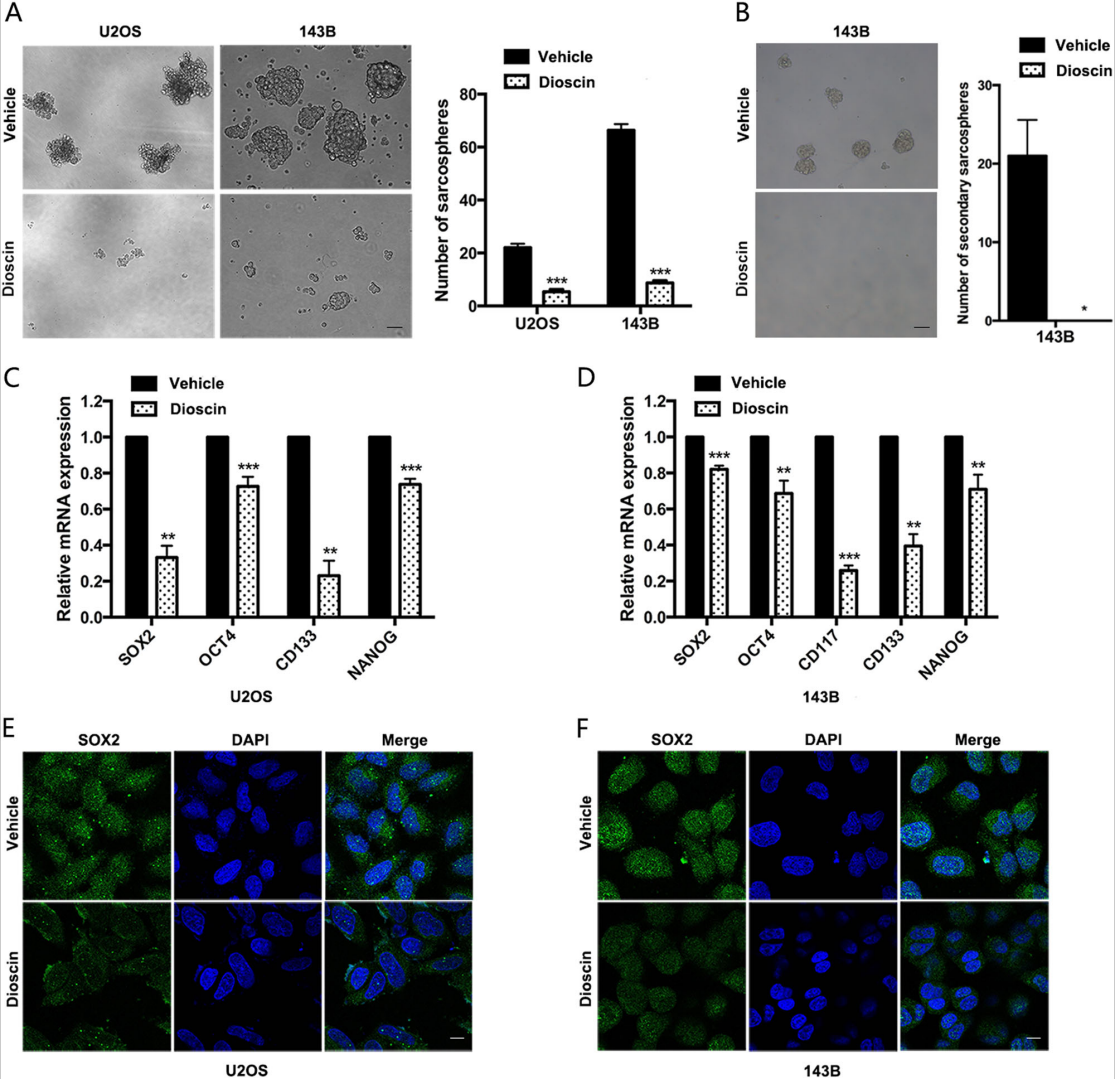
Dioscin suppresses stem-cell-like properties of osteosarcoma cells. **a** Sarcosphere-formation capacity of U2OS and 143B cells was analyzed after 2.5 μm dioscin treatment. Scale bar, 100 μm. **b** Secondary sarcosphere formation capacity of 143B cells was analyzed without further treatment after 2.5 μm dioscin treatment in primary sarcosphere-formation assay. Scale bar, 100 μm. **c**, **d** mRNA expression of stem cell markers (SOX2, OCT4, CD117, CD133, and NANOG) in U2OS and 143B cells treated with vehicle or 2.5 μm dioscin for 48 h was examined by qRT-PCR. **e**, **f** SOX2 protein expression in indicated cells was analyzed by immunofluorescence. Scale bar, 10 μm. Data represent the mean ± SD of three independent experiments. *p < 0.05, **p < 0.01, ***p < 0.001 by two-tailed Student’s *t* test, SPSS 20.0

The treatment failure can be observed in a considerable number of patients who either are not sensitive to chemotherapy or develop drug resistance with current chemotherapy regimens, which would limit the improvement of clinical outcomes^3^. And cancer stem cells (CSCs) have been reported to possess the properties to self-renew and maintain the phenotype of tumor, which may lead to clinical treatment failure^17–19^. The above results have shown the inhibitory effect of dioscin on osteosarcoma both in vitro and in vivo; we sought to investigate whether dioscin could suppress stem-cell-like properties of osteosarcoma cells. Sarcosphere-formation assay showed that dioscin treatment significantly induced smaller and fewer sarcospheres formation in U2OS (*p* < 0.001) and 143B (*p* < 0.001) (Fig. 3a). Furthermore, we performed secondary sarcospheres formation assay to examine whether dioscin affects self-renewal ability of osteosarcoma stem cells. And we found no secondary sarcospheres formation in osteosarcoma cells 143B without further treatment after 2.5 μm dioscin treatment in primary sarcosphere-formation assay, while 143B treated with vehicle could form secondary sarcospheres (*p* < 0.05) (Fig. 3b).

Next, we detected the expression of several stem cell markers after dioscin treatment in U2OS and 143B cells. As shown in Fig. 3c, d, dioscin treatment induced a significant decline in all the stem cell markers, including SOX2, OCT4, CD117, CD133, and NANOG. However, CD117 could not be detected in U2OS in every independent experiment. Furthermore, the protein expression of SOX2, which was reported to maintain the self-renewal of osteosarcoma-initiating cells, significantly decreased after dioscin treatment (Fig. 3e, f).

In summary, dioscin decreases the stem-cell-like population and suppresses stemness properties of osteosarcoma cells. And dioscin negatively regulates self-renewal ability of osteosarcoma stem cells.

### Dioscin inhibits osteosarcoma stem-cell-like properties and tumor growth through repression of Wnt/β-catenin pathway

**Fig. 4:**
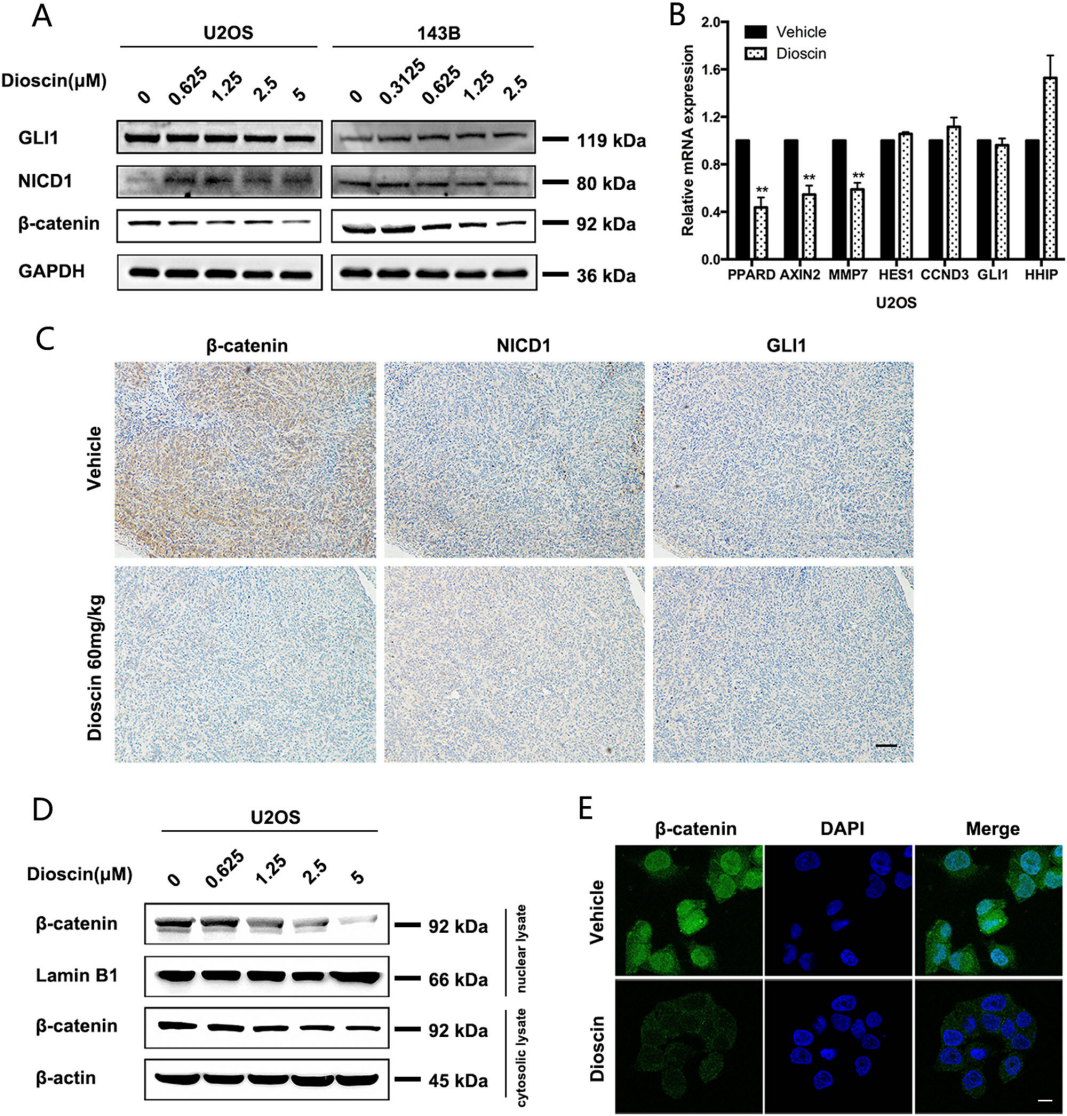
Dioscin targets osteosarcoma-cell-like properties by repression of Wnt/β-catenin pathway. **a** The expression of three critical proteins (β-catenin, NICD1, and GLI1) involved in CSC pathways was examined in U2OS and 143B cells treated with different concentrations of dioscin for 48 h. **b** mRNA expression of target genes of Wnt/β-catenin pathway (PPARD, AXIN2, and MMP7), Notch pathway (HES1 and CCND3), and Hedgehog pathway (GLI1 and HHIP) were determined by qRT-PCR in U2OS cells treated with vehicle or 2.5 μm dioscin for 48 h. **c** IHC staining of β-catenin, NICD1 and GLI1 in tumor samples from mice treated with vehicle or 60 mg/kg dioscin every day. Scale bar, 100 μm. **d** The level of cytosolic and nuclear β-catenin was detected in U2OS cells treated with different concentrations of dioscin for 48 h. **e** β-catenin protein expression was analyzed by immunofluorescence in 143B cells treated with vehicle or 2.5 μm dioscin for 48 h. Scale bar, 10 μm. Data represent the mean ± SD of three independent experiments. **p < 0.01, ***p < 0.001 by two-tailed Student’s *t* test, SPSS 20.0

Dioscin has shown the inhibitory effect on stem-cell-like phenotype of osteosarcoma. To clarify the mechanism of anti-tumor effects induced by dioscin, we mainly focused on the CSC pathways (Wnt, Notch, and Hedgehog)^20–22^. Firstly, we examined the expression of three critical proteins (β-catenin, NICD1, and GLI1) involved in CSC pathways^23–27^. And we found that only β-catenin decreased in a dose-dependent manner after dioscin treatment, while no significant changes could be observed in NICD1 and GLI1 (Fig. 4a). We further detected the expression of some downstream target genes of CSC pathways. Consistently, a significant decline could be found only in target genes of Wnt/β-catenin pathway (PPARD, AXIN2, and MMP7), but not the target genes of Notch pathway (HES1 and CCND3) or Hedgehog pathway (GLI1 and HHIP) (Fig. 4b and Supplementary Figure S1D).

To confirm the above result in vivo, IHC assay for β-catenin, NICD1, and GLI1 in mice tumor samples was performed to analyze the changes in CSC pathways in vivo induced by dioscin treatment. And the results indicated that the tumor treated with dioscin had a significant decrease in the expression of β-catenin, but not NICD1 or GLI1, when compared to the Vehicle group (Fig. 4c).

It has been reported that β-catenin nuclear import is a critical step of Wnt/β-catenin signaling pathway^23,24^. To further verify whether dioscin inhibits osteosarcoma through repression of Wnt/β-catenin pathway, western blot and immunofluorescence were used to detect the level of cytosolic and nuclear β-catenin. As shown in Fig. 4d, e, both cytosolic and nuclear β-catenin reduced after dioscin treatment. More importantly, there was a more significant decrease in nuclear β-catenin.

**Fig. 5:**
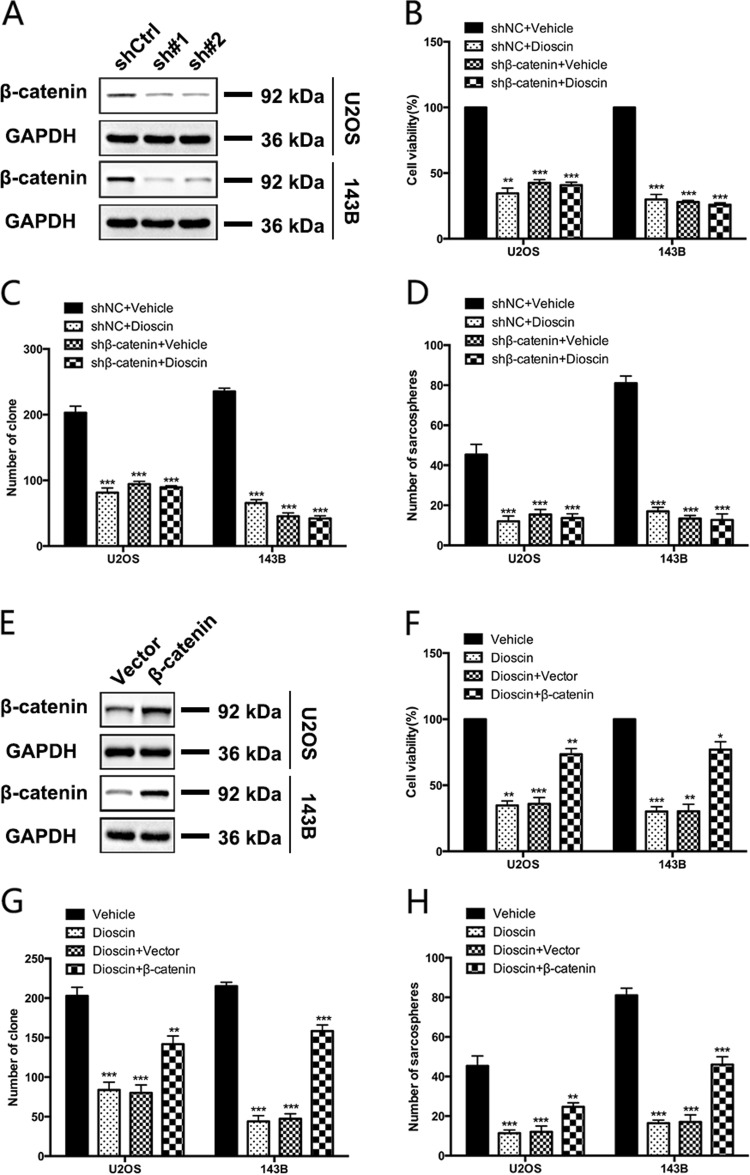
The inhibitory effect of dioscin on osteosarcoma stem-cell-like properties and tumor growth depends on Wnt/β-catenin pathway. **a** The efficiency of β-catenin-shRNA was examined by western blot. **b**−**d** Dioscin has a minimal effect on proliferation (**b**), colony formation (**c**) and sarcospheres formation (**d**) of osteosarcoma cells with β-catenin knockdown. **e** The efficiency of β-catenin-overexpression was examined by western blot. **f**−**h** The impairment of dioscin on proliferation (**f**), colony formation (**g**) and sarcospheres formation (**h**) was partly rescued by overexpressing β-catenin in osteosarcoma cells. Data represent the mean ± SD of three independent experiments. *p < 0.05, **p < 0.01, ***p < 0.001 by two-tailed Student’s *t* test, SPSS 20.0

Next, we explore whether the inhibitory effect of dioscin on osteosarcoma stem-cell-like properties and tumor growth depends on Wnt/β-catenin pathway. As shown in Fig. 5a–d, dioscin had no effect on proliferation, colony formation and sarcospheres formation of osteosarcoma cells with β-catenin knockdown (*p* > 0.05). Besides, the inhibition of dioscin on proliferation, colony formation and sarcospheres formation could be partly rescued by overexpressing β-catenin in osteosarcoma cells (Fig. 5e–h).

Collectively, these results demonstrate that dioscin inhibits osteosarcoma stem-cell-like properties and tumor growth through repression of Wnt/β-catenin pathway.

### β-catenin promotes stem-cell-like properties and tumor growth of osteosarcoma cells

**Fig. 6:**
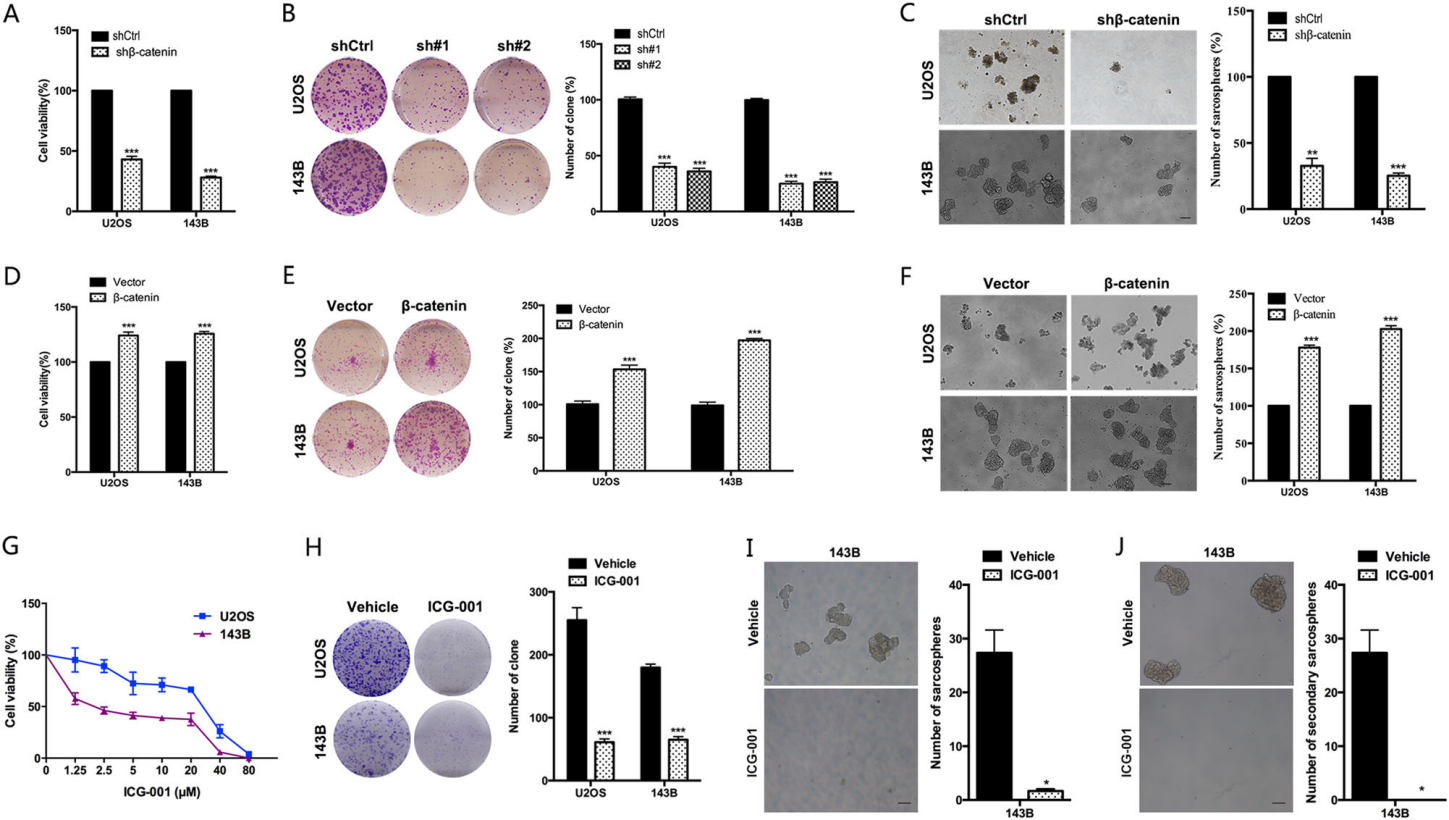
β-catenin promotes the proliferation and stem-cell-like properties in osteosarcoma cells. **a**−**c** The effects of deletion of β-catenin using shRNA on proliferation (**a**), colony formation (**b**) and sphere formation (**c**) were analyzed. **d**−**f** The effects of β-catenin overexpression on proliferation (**d**), colony formation (**e**) and sarcospheres formation (**f**) were analyzed. Scale bar, 100 μm. **g** β-catenin inhibitor ICG-001 inhibits osteosarcoma cells (U2OS, 143B) viability in a dose-dependent manner. Osteosarcoma cells were treated with various concentrations of ICG-001 for 72 h, and the viability of cells was measured by the MTT assay. **h** ICG-001 reduces colony formation of osteosarcoma cells. Colony-formation ability of osteosarcoma cells (U2OS, 143B) was examined after 20 μm ICG-001 treatment for 10 days. **i** Sarcosphere-formation capacity of 143B cells was analyzed after 20 μm ICG-001 treatment. Scale bar, 100 μm. **j** Secondary sarcosphere-formation capacity of 143B cells was analyzed without further treatment after 20 μm ICG-001 treatment in primary sarcosphere-formation assay. Scale bar, 100 μm. Data represent the mean ± SD of three independent experiments. *p < 0.05, **p < 0.01, ***p < 0.001 by two-tailed Student’s *t*test, SPSS 20.0

The above results suggest that β-catenin may be the target of dioscin. Next, we sought to investigate the role of β-catenin in maintaining stem-cell-like properties and tumor growth of osteosarcoma. We found that suppression of β-catenin expression significantly reduced proliferation, colony formation and sarcospheres formation in osteosarcoma cells (Fig. 6a–c). By overexpressing β-catenin, we further confirmed its effect on stem-cell-like properties and tumor growth in osteosarcoma. It could be observed that overexpression of β-catenin increased proliferation, colony formation and sarcospheres formation in osteosarcoma cells (Fig. 6d–f).

Furthermore, two β-catenin inhibitors ICG-001 and XAV-939 were used to examine the role of β-catenin in osteosarcoma. ICG-001 and XAV-939 reduced cell viability of osteosarcoma cell lines U2OS and 143B in a dose-dependent manner (Fig. 6g and Supplementary Figure S1G). And the number and size of colony were greatly reduced in OS cells treated with β-catenin inhibitors ICG-001 and XAV-939 (Fig. 6h and Supplementary Figure S1H).

Besides proliferation suppression, we further found that treatment with β-catenin inhibitors efficiently induced apoptosis in OS cells. The portion of apoptotic cells increased after being treated by 20 μm ICG-001 (Supplementary Figure S1E). And brighter blue staining and typical morphological changes of apoptosis such as the reduction of nuclear size and chromatin condensation were more easily observed in nuclear chromatin of OS cells after 20 μm ICG-001 treatment (Supplementary Figure S1F).

Consistent with the results of knocking down β-catenin, β-catenin inhibitor ICG-001 treatment significantly reduced the size and number of sarcospheres formation in 143B (*p* < 0.05) (Fig. 6i). And we also found no secondary sarcospheres formation in 143B without further treatment after ICG-001 treatment in primary sarcosphere-formation assay, while 143B treated with vehicle could form secondary sarcospheres (*p* < 0.05) (Fig. 6j).

Taken together, these results indicate that β-catenin positively regulates stem-cell-like properties and tumor growth of osteosarcoma.

### Dioscin suppresses Wnt/β-catenin pathway mainly by blocking the phosphorylation of Akt and GSK3

To gain insights on the molecular mechanism by which dioscin suppresses Wnt/β-catenin pathway, we focused on glycogen synthase kinase 3 (GSK3) because GSK3 participates in different signaling pathways, most notably the Wnt/β-catenin pathway in cells^28–31^. Since GSK3 is a kinase within the β-catenin destruction complex, which could phosphorylate Axin-bound β-catenin and the phosphorylated β-catenin is then ubiquitinated and targeted for rapid destruction by the proteasome, preventing activation of β-catenin target genes^32,33^, GSK3 can act as a negative regulator of β-catenin. Moreover, GSK3 was the first Akt substrate reported^34^. Akt is reported to inactivate GSK3 by phosphorylation on an amino-terminal motif conserved in both GSK-3α (S21) and GSK-3β (S9)^28,35–37^. Therefore, we hypothesized that dioscin blocks the phosphorylation of Akt and subsequently activates GSK3 by decreasing the phosphorylation of GSK3, which promotes β-catenin degradation and reduces β-catenin nuclear import.

**Fig. 7:**
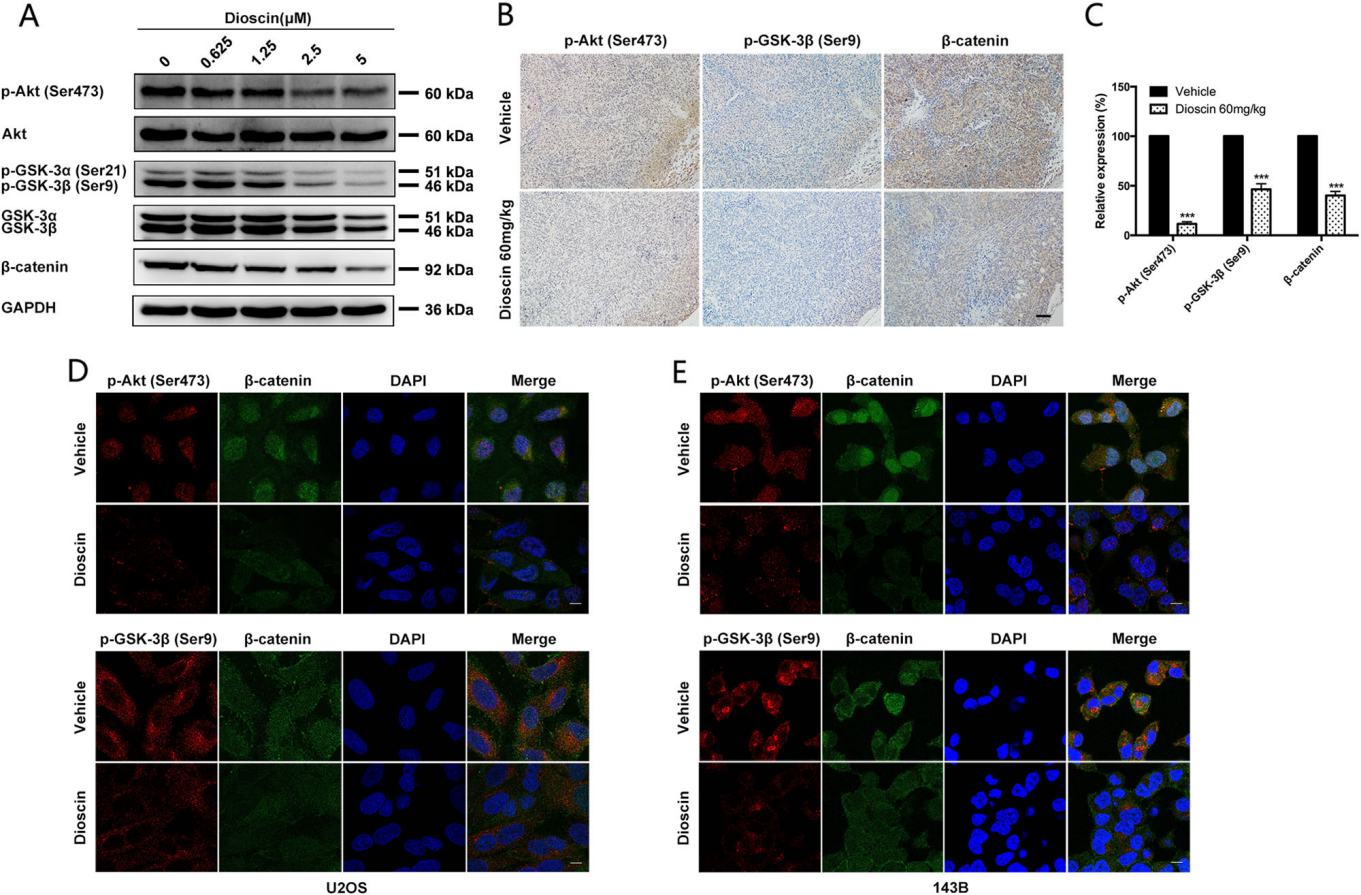
Dioscin suppresses Wnt/β-catenin pathway mainly by blocking the phosphorylation of Akt and GSK3. **a** The level of phosphorylated-Akt (Ser473), total Akt, phosphorylated-GSK3α/β (Ser21/9), total GSK3α/β and β-catenin were examined by western blot in U2OS cells treated with different concentrations of dioscin for 48 h. Dioscin blocked the phosphorylation of Akt and GSK3 in a dose-dependent manner. **b** The level of p-Akt (Ser473), p-GSK3β (Ser9) and β-catenin was detected by IHC staining in same tumor samples of mice treated with vehicle or dioscin 60 mg/kg. Scale bar, 100 μm. **c** The intensity of IHC staining (p-Akt (Ser473), p-GSK3β (Ser9) and β-catenin) was scored. Dioscin 60 mg/kg significantly decreased the level of p-Akt (Ser473), p-GSK3β (Ser9) and β-catenin in vivo. Data represent the mean ± SD of IHC staining scores of eight mice. **d**, **e** The level of p-Akt (Ser473), p-GSK3β (Ser9), and β-catenin was analyzed by immunofluorescence in U2OS and 143B cells treated with vehicle or 2.5 μm dioscin for 48 h. Scale bar, 10 μm. ***p < 0.001 by two-tailed Student’s *t*test, SPSS 20.0

To verify this hypothesis, we investigated whether the phosphorylation of Akt and GSK3 changed after dioscin treatment both in vitro and in vivo. And we found that phosphorylated-Akt (Ser473) and phosphorylated-GSK3α/β (Ser21/9) gradually decreased in a dose-dependent manner after dioscin treatment (Fig. 7a). More importantly, a co-expression pattern was observed in immunofluorescence and IHC assay. As shown in Fig. 7b, c, dioscin treatment induced a significant reduction of phosphorylated-Akt (Ser473), phosphorylated-GSK3β (Ser9) and β-catenin in same mice tumor samples. In addition, phosphorylated-Akt (Ser473) and phosphorylated-GSK3β (Ser9) obviously decreased in osteosarcoma cells treated with dioscin, which was followed by a reduction of β-catenin, especially nuclear β-catenin (Fig. 7d, e).

In summary, dioscin suppresses Wnt/β-catenin pathway mainly by blocking Akt activity, subsequently activating GSK3 and promoting β-catenin degradation.

### Definition of cutoff score for β-catenin expression and its prognostic value in human osteosarcoma patients

**Fig. 8:**
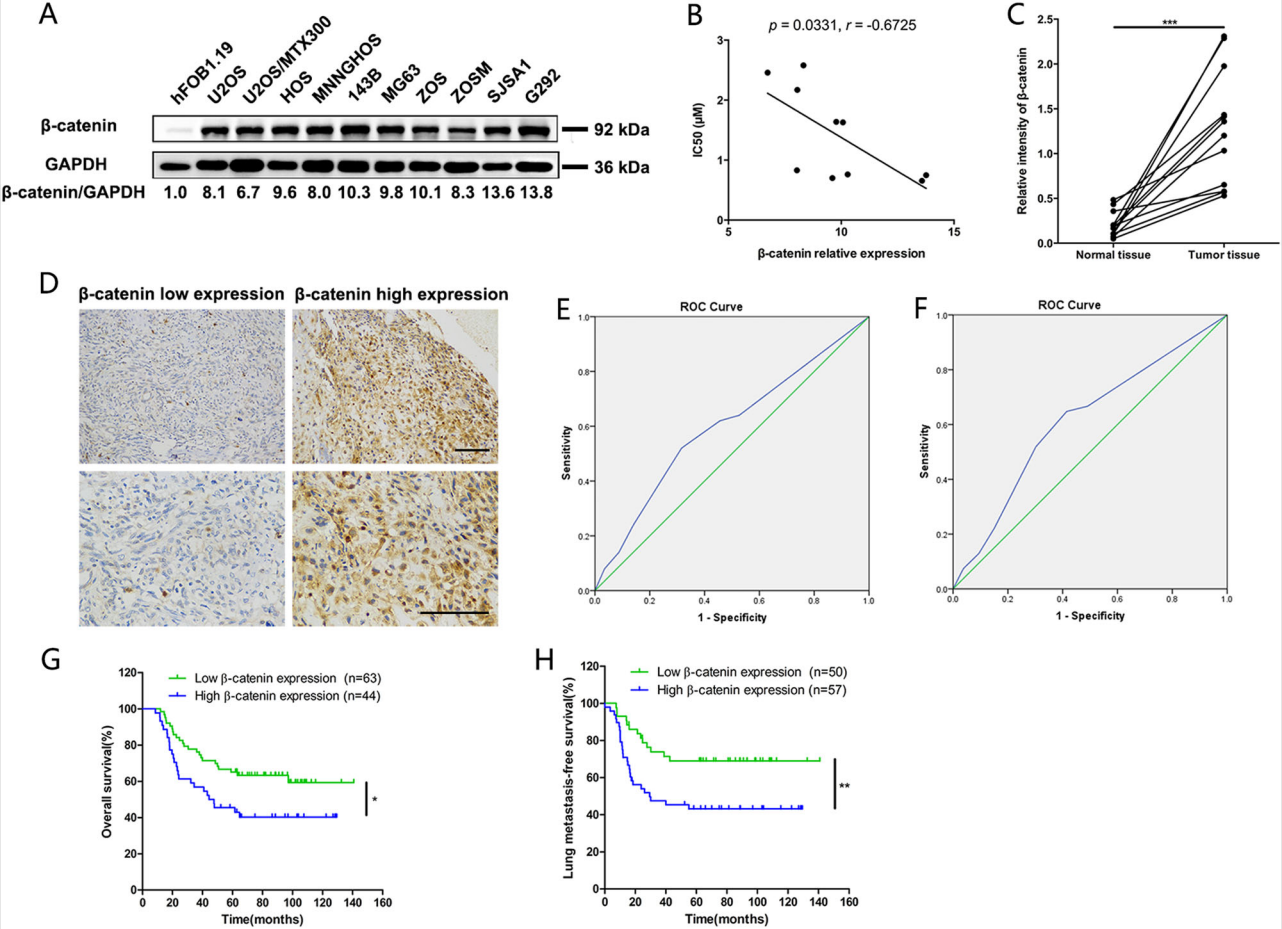
Definition of cutoff score for β-catenin expression and its prognostic value in human osteosarcoma patients. **a** Representative expression pattern of β-catenin protein in osteosarcoma cell lines was shown. The ratio of β-catenin /GAPDH was indicated. **b** β-catenin protein expression was negatively correlated with IC50 (μm) in human osteosarcoma cell lines (Pearson’s correlation analysis, *p* = 0.0331). **c** Protein expression of β-catenin in 12 paired human osteosarcoma tissues and adjacent normal muscle tissues was detected by western blot. Relative intensity of β-catenin normalized to GAPDH was calculated. The expression level of β-catenin in human osteosarcoma tissues was significantly higher than that in normal muscle tissues (Paired *t* test, *p* < 0.001). **d** Immunohistochemical staining was used to detect β-catenin protein expression in human osteosarcoma tissues (*n* = 107). Representative images of low and high β-catenin expression are shown. Scale bars, 100 μm. **e**, **f** Determination of the cutoff score for β-catenin expression in osteosarcoma by receiver operating characteristic (ROC) curves. For survival status and lung metastases status of osteosarcoma patients, the sensitivity and 1-specificity were plotted. The areas under curve (AUC) and the *p* value were indicated. **g** Overall survival was significantly higher in the low-β-catenin-expression group (log-rank test, *p* = 0.0178). **h** The risk of lung metastasis was significantly higher in the high-β-catenin-expression group (log-rank test, *p* = 0.0082)

Based on the above demonstration that β-catenin has an important role in osteosarcoma stem-cell-like properties and tumor growth, representative expression pattern of β-catenin protein in osteosarcoma cell lines and human osteosarcoma tissues was detected. Compared to the normal control human osteoblast hFOB1.19, the expression of β-catenin protein was significantly upregulated in osteosarcoma cell lines (Fig. 8a). Moreover, β-catenin protein expression was negatively correlated with IC50 (μm) in osteosarcoma cell lines (Pearson’s correlation analysis, *p* = 0.0331, Fig. 8b). And expression of β-catenin in 12 paired human osteosarcoma tissues and adjacent normal muscle tissues was also detected by western blot. As shown in Fig. 8c, the expression of β-catenin in human osteosarcoma tissues was significantly higher than that in normal muscle tissues (Paired *t* test, *p* < 0.001).

To further investigate the value of β-catenin as a prognostic marker in osteosarcoma, the clinical significance of β-catenin in primary human osteosarcoma specimens was evaluated. Totally, surgical specimens from 107 cases of osteosarcoma were examined for β-catenin expression (Fig. 8d and Supplementary Table S1).

To better assess the expression of β-catenin in primary human osteosarcoma, ROC curve was employed to define an optimal cutoff value for high β-catenin expression, based on the results of IHC assay and evaluation. As shown in Fig. 8e, f, the areas under curve (AUC) for survival status and lung metastases status were 0.596 and 0.613, respectively (the *p* values were 0.086 and 0.044, respectively). Thus, the cutoff values were selected to achieve the maximum Youden index. After analysis, the cutoff scores for high β-catenin expression were 5.00 for survival status and 3.50 for lung metastases status, respectively. And tumors with scores higher than the obtained cutoff values were considered to have high β-catenin expression.

Based on the definition of cutoff scores, Kaplan−Meier survival analysis revealed that patients with high β-catenin expression (β-catenin IHC score higher than 5.00) had worse overall survival than those with low β-catenin expression (log-rank test, *p* = 0.0178, Fig. 8g). In addition, the risk of lung metastases was significantly higher in the patients expressing high levels of β-catenin (β-catenin IHC score higher than 3.50) than in those expressing low levels of β-catenin (log-rank test, *p* = 0.0082, Fig. 8h). Furthermore, the correlations between β-catenin expression and clinical characteristics are analyzed and presented, which indicated the level of β-catenin could be a potential predictor of prognosis and lung metastasis in osteosarcoma (Table 1).

**Table 1:** The association of β-catenin expression with patient clinicopathological characteristics in 107 osteosarcoma tissues

	Number	β-catenin expression level		*P* value*
		High	Low	
*Age (years)*				0.951
≤20	83	34	49	
>20	24	10	14	
*Gender*				0.501
Male	72	28	44	
Female	35	16	19	
*Location*				0.255
Distal femur	51	23	28	
Proximal tibia	21	10	11	
Proximal humerus	4	1	3	
Proximal fibula	6	0	6	
Others	25	10	15	
*Relapse*				0.366
Yes	8	5	3	
No	99	39	60	
*Lung metastasis*				**0.023**
Yes	54	28	26	
No	53	16	37	
Death				**0.032**
Yes	50	26	24	
No	57	18	39	

They are the p-values that are less than 0.05

*Chi-square test

## Discussion

Osteosarcoma is the most common primary bone tumor, which mainly affects children and adolescents^1^. Combination of multi-agent chemotherapy and aggressive surgery has significantly improved the 5-year survival rate of patients with osteosarcoma from 10 to 70% over the past 30 years^1^. However, although there are some advances in the treatment of osteosarcoma patients, therapies have not improved significantly for recent years^38,39^. It has become an urgent task to identify more effective agents with less severe side effects for osteosarcoma patients. In the present study, we demonstrated that dioscin, a kind of steroidal saponin extracted from *Discorea nipponica Makino*, diminishes osteosarcoma stem-cell-like properties and tumor growth through repression of Akt/GSK3/β-catenin pathway in vitro and in vivo. More importantly, dioscin could be given by oral administration and shows no obvious side effects in vivo, which reflects the potential application value of dioscin in clinics and the great necessity to further develop clinical trials of dioscin.

Recent studies have reported that CSCs possess the properties to self-renew and maintain the phenotype of tumor, which may lead to clinical treatment failure^17–19^. There is increasing amount of evidence that osteosarcoma possesses CSCs, which will have great influence on the design and evaluation of novel targeted treatments for osteosarcoma. The current treatment, combination of chemotherapy and aggressive surgery, can only cure around 70% of osteosarcoma patients because of chemo-resistance^40,41^. And osteosarcoma CSCs are proposed to be responsible for chemo-resistance, which should be considered as an important target for developing novel regimens^40–44^. Therefore, drugs that could directly target osteosarcoma CSCs or increase sensitivity of osteosarcoma CSCs to current chemotherapy regimens could be promising for osteosarcoma patients in clinic. In this study, dioscin possesses the ability to suppress stem-cell-like properties of osteosarcoma cells and significantly decrease osteosarcoma CSCs population, which indicates that dioscin could be an effective regimen for osteosarcoma CSC-targeted therapy. Therefore, dioscin may have the potential to improve the clinical outcomes of osteosarcoma patients.

Over the last two decades, the critical pathways involved in maintaining stem-cell-like properties of CSCs have drawn great interest. Given the importance of three main CSC pathways (Wnt, Notch, and Hedgehog)^20,21^, it is reasonable to develop novel regimens targeting these CSC pathways. Among the main CSC pathways, we found dioscin inhibits osteosarcoma CSCs through repression of Wnt/β-catenin pathway, rather than Notch or Hedgehog pathway. Although controversial conclusions have been made on the role of Wnt/β-catenin pathway in osteosarcoma, there are other studies showing the therapeutic effect of targeting Wnt/β-catenin pathway in osteosarcoma. For example, our previous study^14^ demonstrated that salinomycin targets osteosarcoma CSCs by suppressing the Wnt/β-catenin pathway, which is consistent with the findings in the present study.

Here, we further demonstrated that β-catenin is significantly overexpressed in osteosarcoma cells, compared to the osteoblast hFOB1.19. And a higher expression of β-catenin was detected in osteosarcoma tissues, when compared to the normal tissues. Besides, the IHC assay showed that β-catenin protein is strongly expressed in 41.12% (44/107) clinical samples, and correlates with prognosis of osteosarcoma patients. Collectively, these results suggest that β-catenin could be a promising therapeutic target as well as a significant prognostic marker in osteosarcoma. More importantly, dioscin shows its significant therapeutic effect on osteosarcoma by targeting Wnt/β-catenin pathway, which indicates the clinical application value of dioscin for osteosarcoma patients.

GSK3 could phosphorylate Axin-bound β-catenin and then promote the rapid destruction of phosphorylated β-catenin, preventing the transcription of β-catenin target genes in the nucleus^32,33^. Moreover, Akt is reported to inactivate GSK3 by phosphorylation^28,35–37^. In this study, we demonstrated that dioscin inhibits stem-cell-like properties and tumor growth of osteosarcoma cells through Akt/GSK3/β-catenin signaling. However, how dioscin inhibits the activity of Akt and whether the inhibition is direct needs further study.

In conclusion, the present study provides comprehensive evidence for the inhibition of dioscin on osteosarcoma stem-cell-like properties and tumor growth through repression of Akt/GSK3/β-catenin pathway, which suggests dioscin as a promising therapeutic regimen without obvious side effects for osteosarcoma patients. And this study not only identifies the Akt/GSK3/β-catenin axis as a critical regulator of osteosarcoma CSCs, but also clarifies that β-catenin may be a novel therapeutic target as well as a significant prognostic marker for osteosarcoma clinical treatment. The safety and efficacy of dioscin as a novel drug for osteosarcoma patients will be further evaluated in clinical studies.

## Electronic supplementary material

Supplementary Figure S1

Supplementary Figure Legend

Supplementary Table S1
