# Supplementary figures and images for: Dioscin inhibits stem-cell-like properties and tumor growth of osteosarcoma through Akt/GSK3/β-catenin signaling pathway
Source: Cell Death Dis. 2018 Mar 1;9(3):343. doi: 10.1038/s41419-018-0363-x (PMC5832770; doi:10.1038/s41419-018-0363-x)

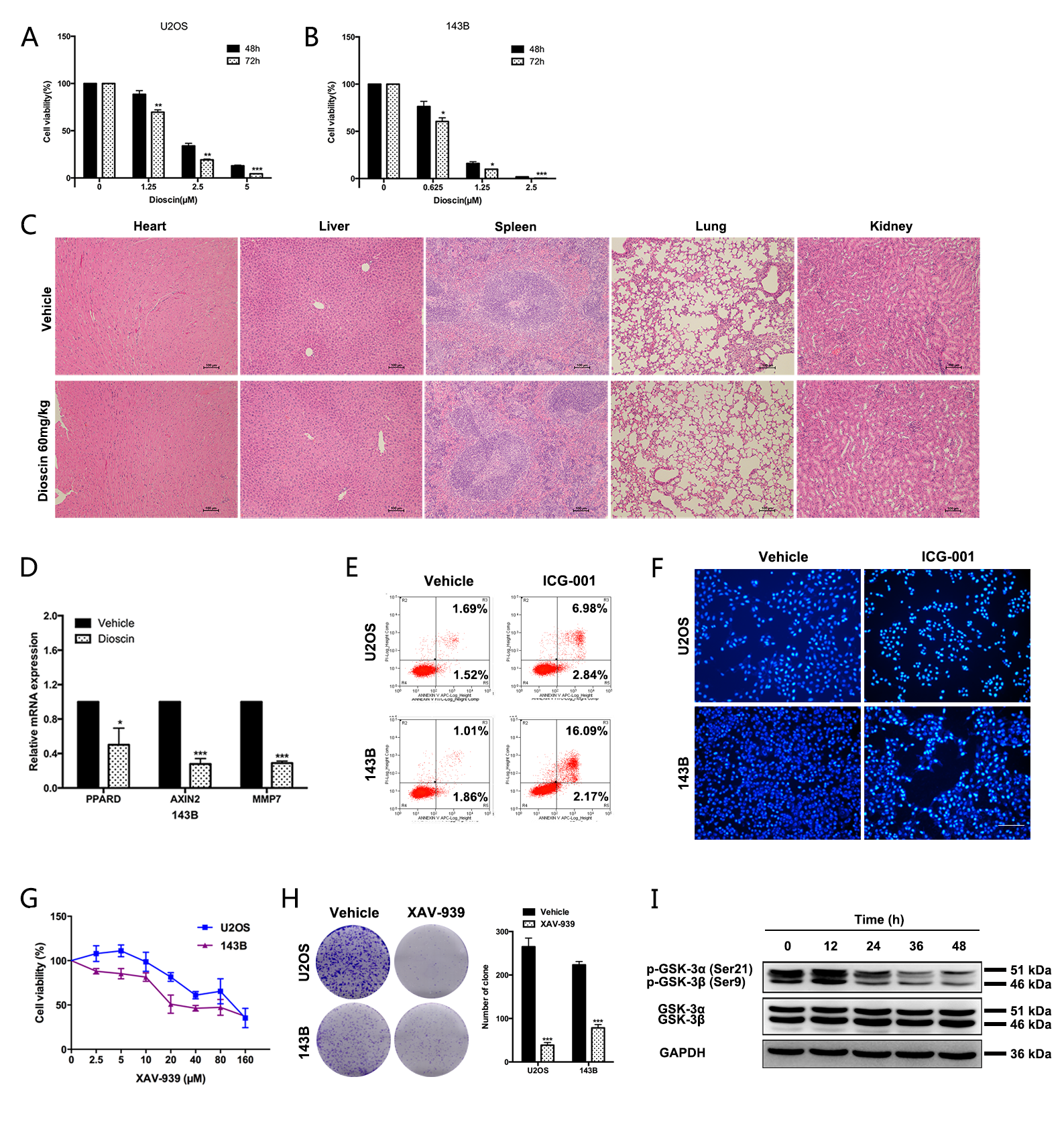

Supplement: Supplementary file 1 — Supplementary Figure S1 [file 41419_2018_363_MOESM1_ESM.tif]
