# Supplementary material for: Dioscin inhibits stem-cell-like properties and tumor growth of osteosarcoma through Akt/GSK3/β-catenin signaling pathway
Source: Cell Death Dis. 2018 Mar 1;9(3):343. doi: 10.1038/s41419-018-0363-x (PMC5832770; doi:10.1038/s41419-018-0363-x)
Supplement: Supplementary file 2 — Supplementary Figure Legend [file 41419_2018_363_MOESM2_ESM.docx]

**Supplementary Figure Legend**

**Supplementary Figure S1.** (A, B), dioscin inhibits osteosarcoma cell (U2OS, 143B) viability in a dose- and time-dependent manner. (C), dioscin did not cause obvious pathologic abnormalities in normal organs of mice. H&E staining of paraffin embedded sections of the heart, liver, spleen, lung and kidney was shown. Scale bar, 100μm. (D), mRNA expression of target genes of Wnt/β-catenin pathway (PPARD, AXIN2 and MMP7) were determined by qRT-PCR in 143B cells treated with vehicle or 2.5μM dioscin for 48h. (E), Annexin V/PI staining of osteosarcoma cells (U2OS, 143B) treated with vehicle or 20μM ICG-001 for 48h was detected and analyzed by flow cytometry. (F), Hoechst staining showed brighter blue staining and typical morphological changes of apoptosis including the reduction of nuclear size and chromatin condensation in nuclear chromatin of U2OS and 143B cells after 20μM ICG-001 treatment for 24 hours. Scale bar, 100μm. (G), β-catenin inhibitor XAV-939 inhibits osteosarcoma cell (U2OS, 143B) viability in a dose-dependent manner. Osteosarcoma cells were treated with various concentrations of XAV-939 for 72 hours, and the viability of cells was measured by the MTT assay. (H), XAV-939 reduces colony formation of osteosarcoma cells. Colony formation ability of osteosarcoma cells (U2OS, 143B) was examined after 20μM XAV-939 treatment for 10 days. (I), dioscin blocked the phosphorylation of GSK3 in a time-dependent manner. Data represent the mean ± SD of 3 independent experiments. **p* < 0.05, ***p* < 0.01, ****p* < 0.001 by two-tailed Student’s *t-* test, SPSS 20.0.
