# Supplementary material for: Dioscin inhibits stem-cell-like properties and tumor growth of osteosarcoma through Akt/GSK3/β-catenin signaling pathway
Source: Cell Death Dis. 2018 Mar 1;9(3):343. doi: 10.1038/s41419-018-0363-x (PMC5832770; doi:10.1038/s41419-018-0363-x)
Supplement: Supplementary file 3 — Supplementary Table S1 [file 41419_2018_363_MOESM3_ESM.docx]

**Supplementary Table S1. Clinical characteristics of 107 osteosarcoma patients**

|  | Total = 107 | Percentage (%) |
| --- | --- | --- |
| Age (years) |  |  |
| Average | 17 |  |
| Range | 6-43 |  |
| Gender |  |  |
| Male | 72 | 67.3 |
| Female | 35 | 32.7 |
| Location |  |  |
| Distal femur | 51 | 47.7 |
| Proximal tibia | 21 | 19.6 |
| Proximal humerus | 4 | 3.7 |
| Proximal fibula | 6 | 5.6 |
| Others | 25 | 23.4 |
| Enneking |  |  |
| IIB | 53 | 49.5 |
| III | 54 | 50.5 |
| Relapse |  |  |
| Yes | 8 | 7.5 |
| No | 99 | 92.5 |
| Lung metastasis |  |  |
| Yes | 54 | 49.5 |
| No | 53 | 50.5 |
| Death |  |  |
| Yes | 50 | 46.7 |
| No | 57 | 53.3 |
